# Synergetic cytotoxic activity toward breast cancer cells enhanced by the combination of Antp-TPR hybrid peptide targeting Hsp90 and Hsp70-targeted peptide

**DOI:** 10.1186/1471-2407-14-615

**Published:** 2014-08-26

**Authors:** Tomohisa Horibe, Aya Torisawa, Masayuki Kohno, Koji Kawakami

**Affiliations:** Department of Pharmacoepidemiology, Graduate School of Medicine and Public Health, Kyoto University, Yoshida Konoecho, 606-8501 Sakyo-ku, Kyoto, Japan

**Keywords:** Client proteins, Heat shock protein 90, Heat shock protein 70, Hybrid peptide, Molecular chaperone, Breast cancer, Bioluminescence

## Abstract

**Background:**

Heat shock protein (Hsp) 90 and Hsp70 are indispensable for cell survival under conditions of stress. They bind to client proteins to assist in protein stabilization, translocation of polypeptides across the cell membrane, and recovery of proteins from aggregates in the cell. Therefore, these proteins have recently emerged as important targets in the treatment of cancer. We previously reported that the newly designed Antp-TPR hybrid peptide targeting Hsp90 induced cytotoxic activity to cancer cells both *in vitro* and *in vivo*.

**Methods:**

To further improve the cytotoxic activity of Antp-TPR toward cancer cells, we investigated the effect of a Hsp70-targeted peptide, which was made cell-permeable by adding the polyarginine with a linker sequence, on the cytotoxic activity of Antp-TPR in breast cancer cell lines.

**Results:**

It was revealed that Antp-TPR in the presence of a Hsp70-targeted peptide induced effective cytotoxic activity toward breast cancer cells through the descrease of Hsp90 client proteins such as p53, Akt, and cRaf. Moreover, the combined treatment with these peptides did not induce the up-regulation of Hsp70 protein, as determined by western blotting, a promoter assay using a luminometer, and single-cell level imaging with the LV200 system, although a small-molecule inhibitor of Hsp90, 17-allylamino-demethoxygeldanamycin (17-AAG), did induce the up-regulation of this protein. We also found that treatment with Antp-TPR, Hsp70-targeted peptide, or a combination of the two did not induce an increase in the glutathione concentrations in the cancer cells.

**Conclusion:**

These findings suggest that targeting both Hsp90 and Hsp70 with Antp-TPR and Hsp70-targeted peptide is an attractive approach for selective cancer cell killing that might provide potent and selective therapeutic options for the treatment of cancer.

**Electronic supplementary material:**

The online version of this article (doi:10.1186/1471-2407-14-615) contains supplementary material, which is available to authorized users.

## Background

Breast cancer is the most frequently diagnosed cancer and the leading cause of cancer deaths in woman worldwide, accounting for 23% of new cancer cases and 14% of cancer deaths in 2008, although screenings and treatments for the cancer have been recently improved [1]. Since breast cancer is a clinically heterogeneous disease with multiple genetic abnormalities, targeting a single pathway in cancer cells by inhibiting the activity of a single component is not likely to be effective in the long term treatment for the cancer. Therefore, identifying molecular targets with the potential to modulate multiple components of several signaling pathways in the cancer cells will be indispensable for the development of novel breast cancer therapies. In this regard, heat shock protein (Hsp) 90 has attracted considerable attention in recent years as a potential therapeutic target for the identification and development of next-generation anticancer drugs [2]. Several Hsp90 inhibitors such as 17-allylamino-demethoxygeldanamycin (17-AAG; KOS-953) and PU-H71 are recently reported candidates for targeted or combination breast cancer therapies [3–5].

Hsp90, which is an abundant cytosolic molecular chaperone found within multimeric chaperone complexes known to mediate cellular protein homeostasis [2], assists in the correct folding of more than 300 proteins (called as client proteins) [6], including transmembrane tyrosine kinase such as epidermal growth factor receptor 1 (EGFR) and 2 (Her2), metastable signaling proteins such as Akt/PKB and cRaf, mutated signaling proteins such as p53 and v-Src, chimeric signaling proteins such as Bcr-Abl, cell-cycle regulators such as cyclin-dependent kinase 4 (Cdk4) and 6(Cdk6), and steroid receptors such as androgen, estrogen, and progesterone receptors [6–10]. Thus, Hsp90 plays a unique role in cellular homeostasis and has consequently emerged as a promising anticancer target [2].

On the other hand, Hsp70 binds to hydrophobic polypeptide sequences in newly synthesized proteins and partially folded substrates in the cells, thereby directing them to particular cell fates, although Hsp90 has a more restricted repertoire of client proteins [11]. Thus, the inhibition of Hsp70 is another emerging strategy in cancer therapy, and recent genetic and biochemical studies have supported the discovery of Hsp70 inhibitors with potential use as single agents or in combinations to boost the efficacy of conventional chemotherapeutic and molecular targeted drugs, including Hsp90 inhibitors [11]. However, no specific inhibitors of this protein are clinically available at present. Rérole *et al*. recently identified multiple peptide aptamers that bind to Hsp70 and demonstrated that two of them specifically inhibit chaperone activity while also increasing the sensitivity of cancer cells to apoptosis induced by anticancer drugs [12]. In addition, these peptides specifically inhibited Hsp70 and induced the regression of subcutaneous tumors *in vivo* after local and systemic injection [12].

We previously reported that the newly designed Antp-TPR hybrid peptide inhibits the interaction of Hsp90 with the tetratricopeptide repeat 2A domain of p60/Hsp-organizing protein (Hop) [13]. Antp-TPR has cytotoxic activity toward cancer cells through the decrease of Hsp90 client proteins *in vitro* and to induce effective antitumor activity in a xenograft model of human pancreatic cancer in mice *in vivo* [13]. It was also demonstrated that Antp-TPR does not induce up-regulation of Hsp90, Hsp70, and Hsp27 proteins, whereas 17-AAG does [14]. However, the cytotoxic activity of Antp-TPR toward cancer cells was not affected in the presence of 2-phenylethynesulfonamide, which was recently introduced as a small-molecule inhibitor of Hsp70 [15, 16], although the cytotoxic activity of 17-AAG was increased under this condition. These findings prompted us to examine the combination of Antp-TPR with peptides or small compounds targeting Hsp70 in order to further improve the cytotoxic activity of Antp-TPR toward cancer cells. To further clarify the role of Hsp90 targeting by Antp-TPR in cancer treatment, it is important to address the synergetic effect of combination therapy with Hsp70-targeting peptides on the cytotoxic activity of Antp-TPR toward cancer cells. Here we report the effective cytotoxic activity toward breast cancer cells of Antp-TPR in the presence of a Hsp70-targeting peptide. We also show that treatment with a combination of these peptides does not induce up-regulation of Hsp70 or glutathione (GSH) in cancer cells.

## Methods

### Materials

Anti-Hsp90, anti-Hsp70, and anti-Hsp27 antibodies were purchased from Stressgen Bioreagents (Ann Arbor, MI, USA). Anti-c-Raf, anti-Akt, and anti-K48 linkage-specific polyubiquitin antibodies were purchased from Cell Signaling Technology (Danvers, MA, USA). Anti-p53 and anti-βactin antibodies were purchased from Sigma (St Louis, MO, USA). Anti-polyubiquitinylated protein antibody was purchased from Merck Millipore (Billerica, MA, USA). 17-AAG was purchased from InvivoGen (San Diego, CA, USA). Other reagents were obtained mostly from Nacalai Tesque (Kyoto, Japan). All reagents were of reagent grade.

### Cells and cell culture

Three human breast cancer cell lines (MDA-MB-231, BT20, and BT474) and human mammary epithelial cell (MCF-10A) were purchased from the American Type Culture Collection (Manassas, VA, USA). Human breast adenocarcinoma cell line MDA-MB-361 was purchased from the European Collection of Cell Culture (Salisbury, UK). Cells were cultured in RPMI-1640 (MDA-MB-231, BT20, BT474, and MDA-MB-361 cells) supplemented with 10% fetal bovine serum or DMEM/F-12 medium (MCF-10A cells) supplemented with 100 ng/ml cholera toxin solution, 10 μg/ml insulin from bovine pancreas, 0.5 mg/ml hydrocortisone, 20 ng/ml epidermal growth factor, and 5% horse serum, containing 100 μg/ml penicillin and 100 μg/ml streptomycin at 37°C in an atmosphere of 5% CO_2_/95% air.

### Peptide synthesis

Peptides were synthesized by the American Peptide Company (Sunnyvale, CA, USA) and Sigma. Antp-TPR (RQIKIWFQNRRMKWKKKAYARIGNSYFK) was made as described previously [13, 14]. The Hsp70-targeted peptide (YCAYYSPRHKTTF) [12] was made cell-permeable by adding helix III of the cell-penetrating antennapedia homeodomain sequence (Antp) [17] or polyarginine (R11; RRRRRRRRRRR) (italic) [18, 19] with a linker sequence of triple glycine (GGG) as follows: *RQIKIWFQNRRMKWKK*GGGYCAYYSPRHKTTF (Antp-Hsp70) or *RRRRRRRRRRR*GGGYCAYYSPRHKTTF (R11-Hsp70). The scrambled peptide for Hsp70 (TPTYRASCFYHYK) was also hybridized by the R11 peptide with a linker sequence as follows: *RRRRRRRRRRR*GGGTPTYRASCFYHYK (R11-Hsp70scramble). All peptides were dissolved in sterile water and the aliquots of peptide solutions were stored at -20°C until use.

### Western blotting

Western blotting was carried out as described previously [14]. Briefly, protein extracts were prepared from cells lysed with reporter lysis buffer (Promega, Madison, WI, USA), separated by SDS-PAGE and transferred to nitrocellulose filters using the iBlot system (Invitrogen, Carlsbad, CA, USA) according to the manufacturer’s protocol. Quenched membranes were probed with antibodies and analyzed using an enhanced reagent of Chemi-Lumi One Super (Nacalai Tesque) with a LAS-3000 LuminoImage analyzer (Fujifilm, Tokyo, Japan). Densitometric analysis was performed by Multi Gauge software V3.0 (Fujifilm) using the bands obtained from western blotting, in which actin as the loading control was used for normalizing of each bands.

### Cell viability assay

Cell viability was determined by WST-8 assay as described previously [20]. Briefly, cells were seeded onto 96-well plates at 2000–3000 cells/well overnight. After incubating with the test peptides, the cell viability assay was carried out using Living Cell Count Reagent SF (Nacalai Tesque) according to the manufacturer’s protocol. Absorbance was measured at 450 nm using a 96-well microplate reader (GE Healthcare Bioscience, Piscataway, NJ).

### Fluorescence microscopy

Fluorescence images were obtained by Olympus FV1000 confocal laser scanning microscopy (Olympus, Tokyo, Japan) as described previously [20]. Briefly, BT20 cells were plated in a glass-bottomed dish and small aliquots of labeled peptides Antp-TPR-TAMRA (Invitrogen) and R11-Hsp70-FITC (Invitrogen) (where TAMRA and FITC are carboxytetramethylrhodamine and fluorescein isothiocyanate, respectively) were added directly at a final concentration of 10 μM.

### Flow cytometry assay

Flow cytometry assay was performed as described previously [20]. Briefly, after the treatment of cancer or normal cells with or without Antp-TPR-TAMRA in the presence or absence of R11-Hsp70 or R11-Hsp70 scramble peptide, or R11-Hsp70-FITC, cells were collected and washed twice with PBS. The cell pellets were resuspended, and then flow cytometry analysis was performed by FACSCalibur (BD Bioscience, San Jose, CA, USA). Data were analyzed using CellQuest Software (BD Bioscience). Annexin V and PI staining was performed using Annexin V-Fluorescein Staining Kit (Wako Chemicals, Osaka, Japan) by multiparametric flow cytometry assay as described previously [20].

### Reporter assay

A reporter assay was carried out as described previously [21]. Briefly, BT20 cells were transfected using Lipofectamine LTX (Invitrogen) with firefly luciferase-containing reporter plasmids of the Hsp70 promoter (pHsp70Pro-Luc), in which the Hsp70 promoter region (-637 to +165) was cloned into the *Bgl*II-*Hind*III sites of the pGL4.14 vector (Promega) using 5′- CACAATCAATCAGATC***T***CTACTGGCTCACCTAGTC-3′ and 5′- GATCCGCGAGAA***A***AGCT***T***GGTCCTTCCGGACGCCG-3′ as the upper and lower primers, respectively (the mutated nucleotides for introducing into *Bgl*II and *Hind*III are italic). *Renilla* luciferase-containing plasmid pRL-SV40 (Promega) was used as an internal control. The relative activity of firefly luciferase to *Renilla* luciferase activity was determined in triplicate (means ± SD) using the Dual-Glo Luciferase Assay System (Promega).

### Bioluminescence imaging

Stably transfected BT20 cells with pHsp70Pro-Luc were prepared after transient transfection with Lipofectamine LTX according to the manufacturer’s protocol in a selective medium containing 200 μg/ml hygromycine B (Nacalai Tesque). Luminescence images at the single-cell level were obtained using the LV200 luminescence imaging system (Olympus) as described previously [22, 23]. Briefly, the dish was kept at 37°C in a humidified chamber and images were taken with a 40× objective at 5-min intervals with an exposure of 10 s while observing promoter activity after the addition of D-luciferin (Promega) at a final concentration of 500 μM. Data analysis was performed using AQUACOSMOS ver 2.6 software (Hamamatsu Photonics, Shizuoka, Japan).

### Measurement of ATP dynamics

Cellular ATP dynamics were measured on single-cell imaging using the LV200 imaging system as described previously [22]. Briefly, BT20 cells were transiently transfected with firefly luciferase-containing reporter plasmids of the cytomegalovirus promoter pGL4.50 (Promega), and bioluminescence images were obtained as mentioned above after treatment with or without Antp-TPR, R11-Hsp70, or a combination of these peptides.

### GSH assay

The GSH assay was performed after treatment with or without 17-AAG, Antp-TPR, R11-Hsp70, or a combination of these peptides using the GSH-Glo assay kit (Promega) according to the manufacturer’s protocol. Total luminescence intensity obtained with a luminometer was normalized to the total protein concentration of each sample determined spectrophotometrically in a NanoDrop 1000 (Thermo Fisher Scientific Inc. Waltham, MA).

### Statistical analysis

Data are expressed as means ± SD. Significance was determined using Student’s t-test and set at P < 0.05.

## Results

### Increased cytotoxic activity toward breast cancer cells of Antp-TPR in the presence of Hsp70-targeted peptide

**Figure 1:**
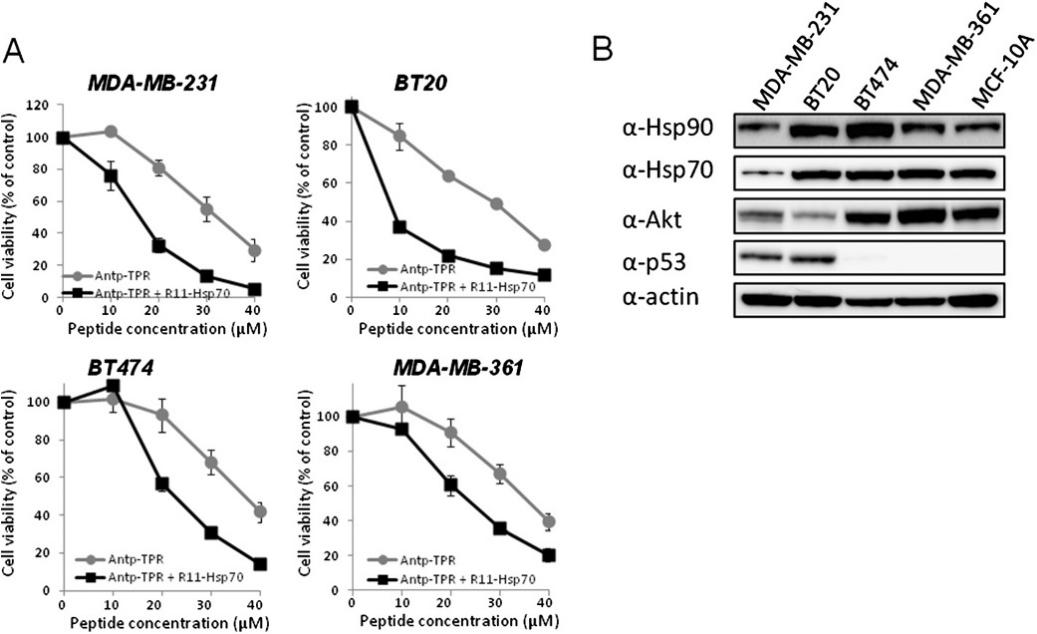
**Increase in the cytotoxic activity toward breast cancer cells of Antp-TPR hybrid peptide in the presence of heat shock protein (Hsp) 70-targeted peptide. (A)** Viability of four breast cancer cell lines (MDA-MB-231, BT20, BT474, and MDA-MB-361) treated with Antp-TPR hybrid peptide in the presence or absence of R11-Hsp70 peptide. Cells were incubated with Antp-TPR at the indicated concentrations in the presence or absence of R11-Hsp70 (10 μM) for 24 h and analyzed for cell viability as described in the Materials and Methods section. Data are expressed as the means ± SD from triplicate determinations. **(B)** Analysis of Hsp90, Hsp70, Akt, and p53 expression in breast cancer and normal cell lines. Cell extracts from the indicated breast cancer and normal cell lines were examined for Hsp90, Hsp70, Akt, and p53 expression by western blotting with corresponding antibodies. β-Actin was used as the loading control. Bands were visualized by chemiluminescence as described in the Materials and Methods section.

First we examined the effect of the Hsp70-targeted peptide [12], which was made further cell-permeable by the addition of Antp (Antp-Hsp70) or R11 (R11-Hsp70), on the cytotoxic activity of Antp-TPR toward cancer cells. The cytotoxic activity of Antp-TPR in the presence of R11-Hsp70 was higher and more effective than that in the presence of Antp-Hsp70 (data not shown). The cytotoxic activity of R11-Hsp70 alone toward MDA-MB-231 and BT20 cells was weaker than that of Antp-TPR alone, and 10 μM R11-Hsp70 barely reduced cell viability (Additional file 1A). However, the cytotoxic activity of Antp-TPR toward breast cancer cells was effectively increased in a concentration-dependent manner in the presence of R11-Hsp70 (Figure 1A). In contrast, no effective increase in the cytotoxic activity of Antp-TPR toward cancer cells was observed in the presence of R11-Hsp70scramble (Additional file 1B). It was also observed that the cytotoxic activity of both Antp-TPR alone and Antp-TPR in the presence of R11-Hsp70 toward normal mammary epithelial cells (MCF-10A) was less than that of these peptides against cancer cell lines, and that R11-Hsp70 did not affect the cytotoxic activity of 17-AAG (Additional file 1C). As shown in Table 1, the IC_50_ values of Antp-TPR alone toward the MDA-MB-231, BT20, BT474, and MDA-MB-361 cell lines were reduced from 26–34 μM to 8–23 μM in the presence of R11-Hsp70, a respective IC_50_ change of 3.1- to 1.4-fold. These results indicate that the Hsp70-targeted peptide can effectively increase the cytotoxic activity of Antp-TPR toward cancer cells. When we examined the endogenous expression levels of Hsp90, Hsp70, Akt, and p53 in the breast cancer and normal cell lines, the expression levels of Hsp90 and Hsp70 in these cell lines were equally unremarkable, except for those in the MDA-MB-231 cells, but the expression levels of Akt and p53 were obviously different among these cell lines (Figure 1B).

**Table 1:** **Inhibitory concentration (IC**_**50**_ **) of Antp-TPR or the combination of Antp-TPR and R11-Hsp70**

		IC _50_(μM)*	
	Antp-TPR	Antp-TPR + R11-Hsp70**	Ratio (Antp-TPR / Antp-TPR + R11-Hsp70
MDA-MD-231	31.2 ± 3.0	16.1 ± 1.0	1.9
BT20	26.1 ± 3.0	8.5 ± 1.0	3.1
BT474	34.1 ± 2.7	23.6 ± 1.1	1.4
MDA-MD-361	33.8 ± 2.6	23.1 ± 2.2	1.5

*Results are the mean of three independent observations each performed in triplicate.

**IC50 of Antp-TPR in the presence of R11-Hsp70 (10 μM).

### Time-dependent synergetic cytotoxic activity of Antp-TPR in the presence of R11-Hsp70

**Figure 2:**
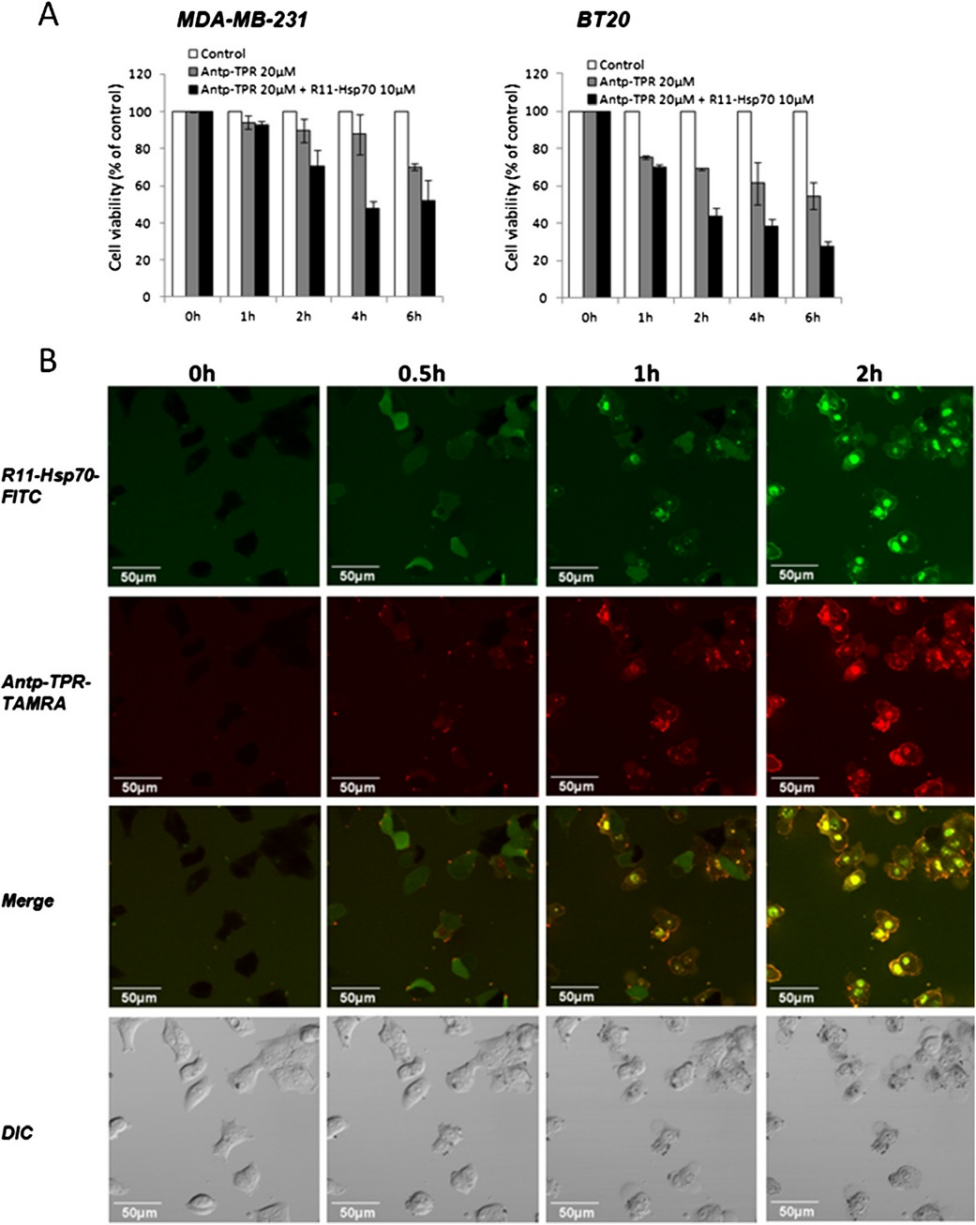
**Time course of synergetic cytotoxic activity toward and intracellular penetration of Antp-TPR in cancer cells in combination with R11-Hsp70. (A)** Time course of cell viability after treatment with Antp-TPR in the presence or absence of R11-Hsp70. MDA-MB-231 and BT20 cells were treated with Antp-TPR (20 μM) in the presence or absence of R11-Hsp70 (10 μM) for the indicated times and analyzed for cell viability by WST-8 assay. Data are expressed as the means ± SD from triplicate determinations. **(B)** Intracellular penetration of Antp-TPR and R11-Hsp70. BT20 cells were incubated with Antp-TPR (10 μM) labeled with carboxytetramethyl rhodamine (TAMRA; Antp-TPR-TAMRA) and R11-Hsp70 (10 μM) labeled with fluorescein isothiocyanate (FITC; R11-Hsp70-FITC) for the indicated times. Cells were analyzed by differential interference contrast (DIC) or fluorescence (FITC-green or TAMRA-red), or as merged images (FITC-green and TAMRA-red). All images were obtained by confocal microscopy as described in the Materials and Methods section. All scale bars are 50 μm.

We next examined the rate of Antp-TPR-mediated cancer cell killing in the presence of R11-Hsp70. As shown in Figure 2A, synergetic cytotoxic activity of Antp-TPR with R11-Hsp70 was observed in a time-dependent manner. Exposure of BT20 cells to 20 μM Antp-TPR with 10 μM R11-Hsp70 for 6 h was sufficient to induce high levels of cytotoxic activity (approximately 70% decrease in cell viability) (Figure 2A). Confocal laser scanning microscopy revealed penetration of the cancer cells by Antp-TPR and R11-Hsp70 labeled with TAMRA and FITC, respectively, in a time-dependent manner (Figure 2B); this indicated the synergetic effect of Antp-TPR in a time-dependent manner.

### Decrease of Hsp90 client proteins after treatment with Antp-TPR in the presence of R11-Hsp70

**Figure 3:**
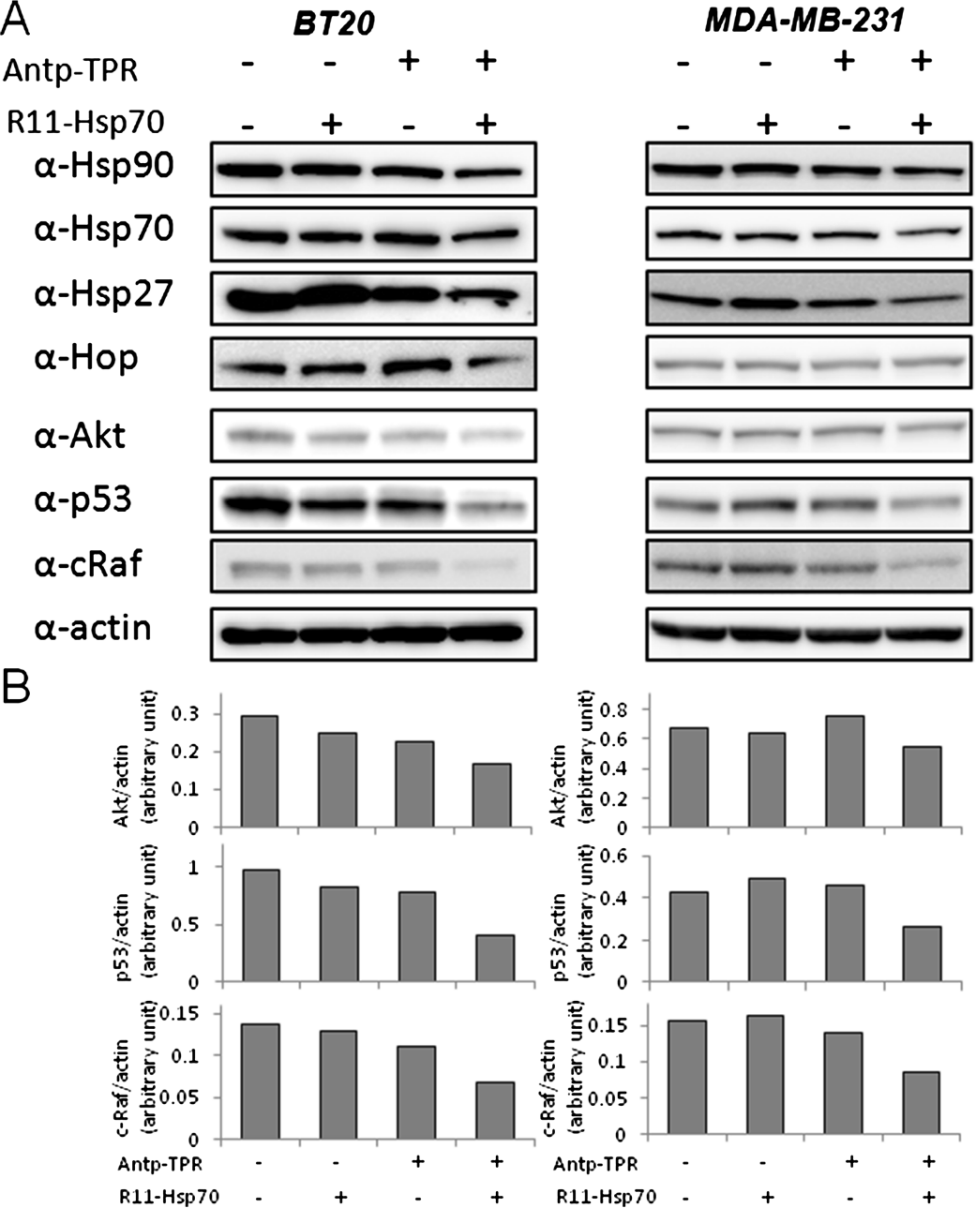
**Decrease of Hsp90 client proteins after treatment with Antp-TPR in the presence of R11-Hsp70 in BT20 and MDA-MB-231 cells. (A)** BT20 and MDA-MB-231 cells were treated with or without Antp-TPR (20 μM), R11-Hsp70 (10 μM), or a combination of these peptides for 18 h and examined by western blotting for the expression of Hsp90, Hsp70, Hsp27, Hop, Akt, p53, cRaf, and β-actin using corresponding antibodies. β-Actin was used as the loading control. Bands were visualized by chemiluminescence. **(B)** Densitometric analysis of Hsp90 client proteins (Akt, P53, and c-Raf) was performed using Multi Gauge software as described in the Materials and Methods section.

We previously demonstrated that Antp-TPR induced cancer cell death through the decrease of Hsp90 client proteins such as Akt, p53, and cRaf in cancer cells [13, 14]. These findings prompted us to investigate the effect of R11-Hsp70 on the decrease of Hsp90 client proteins by Antp-TPR. As shown in Figure 3A and B, treatment of BT20 and MDA-MB-231 breast cancer cells with Antp-TPR in the presence of R11-Hsp70 effectively decreased the expression of Hsp90 client proteins, including Akt, p53, and cRaf, compared with that of Antp-TPR or R11-Hsp70 alone, or untreated control cells, although 10 μM of R11-Hsp70 alone caused the slight decrease of these proteins in BT20 cells, and high concentration of R11-Hsp70 (40 μM) alone also caused the decrease of expression levels of Hsp90, Hsp70, Akt, and p53 proteins in BT20 cells (Additional file 2). When we examined by western blotting the levels of polyubiquitin proteins and K48-linked-polyubiquitin proteins in cancer cells after treatment with these peptides, an increase of several bands of proteins located at approximate 40–48 kDa, which were detected by specific antibody against K48-linked-polyubiqutin proteins was observed in a time-dependent manner after treatment with Antp-TPR in the presence of R11-Hsp70, compared with the other treatments (Additional file 3). Furthermore, treatment with Antp-TPR in the presence of R11-Hsp70 did not induce up-regulation of Hsp90, Hsp70, Hsp27, or Hop proteins in these cancer cells (Figure 3A). These results indicate that Antp-TPR in the presence of R11-Hsp70 induces an effective decrease of Hsp90 client proteins and does not induce up-regulation of heat shock proteins.

### Effect of treatment with Antp-TPR in the presence of R11-Hsp70 on the expression of Hsp70

**Figure 4:**
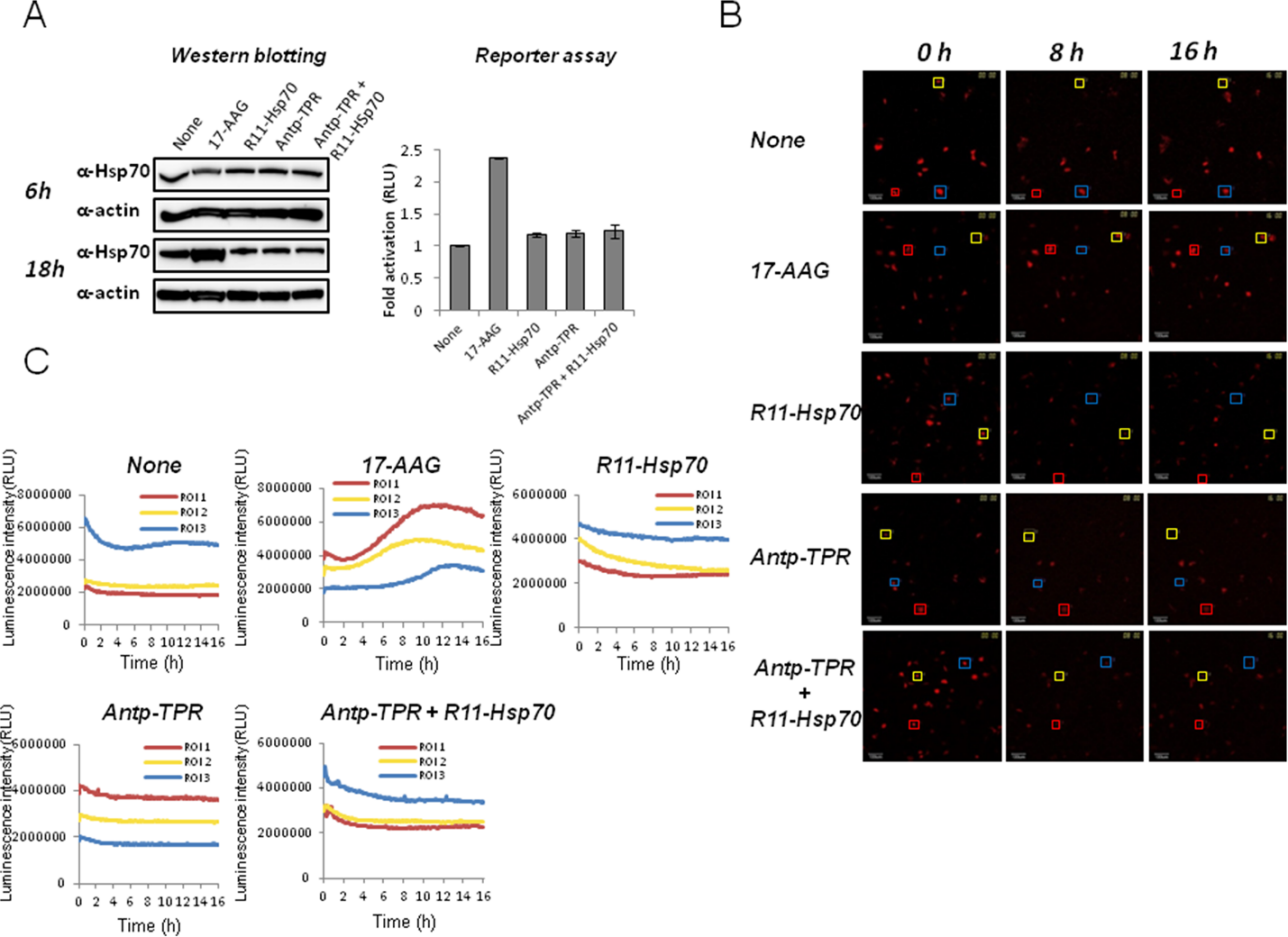
**Effect of treatment with Antp-TPR in the presence of R11-Hsp70 on the expression of Hsp70. (A)** BT20 cells were treated with or without Antp-TPR (20 μM), R11-Hsp70 (10 μM), or a combination of these peptides for 6 h or 18 h and examined by western blotting for the expression of Hsp70 and β-actin using corresponding antibodies (left panel). β-Actin was used as the loading control. BT20 cells were transiently transfected with pHsp70Pro-Luc, and activation of the Hsp70 promoter was assessed by promoter assay using a luminometer 18 h after the treatment with or without Antp-TPR, R11-Hsp70 or a combination of these peptides (right graph). **(B)** Luminescence images (shown in red) of stably transfected BT20 cells with pHsp70Pro-Luc captured by the LV200 system using an exposure of 10s at 0 h, 8 h, and 16 h after treatment with or without Antp-TPR, R11-Hsp70, or a combination of these peptides. Squares in the luminescence images indicate the region of interest (ROI), in which the luminescence intensity was measured for time-lapse analysis at the single-cell level. All scale bars are 100 μm. **(C)** Time-course analysis of Hsp70 promoter activation on single-cell imaging. Stably transfected BT20 cells with pHsp70Pro-Luc were treated with or without Antp-TPR, R11-Hsp70, or a combination of these peptides, and time-course analysis was performed using the LV200 system as described in the Materials and Methods section. In all experiments, 0.5 μM 17-allylamino-demethoxygeldanamycin (17-AAG) was used as a positive control for the up-regulation of Hsp70 in cancer cells.

Conventional Hsp90 inhibitors including 17-AAG induce compensatory up-regulation of Hsp70 that likely correlates with decreased anticancer activity [24, 25], since Hsp70 is also molecular chaperone, can bind to a wide variety of newly synthesized proteins, and then assist the folding of their proteins in the cells [11]. We previously demonstrated that Antp-TPR barely affected the transcription levels of Hsp70 [14]. In this study, we examined the effect of treatment with Antp-TPR in the presence of R11-Hsp70 on cancer cells on the expression of Hsp70. As shown in Figure 4A, neither the treatment with Antp-TPR in the presence or absence of R11-Hsp70 or R11-Hsp70 alone induced up-regulation of Hsp70 or the promoter activity of this protein, whereas 17-AAG induced both. Activation of the Hsp70 promoter after treatment with 17-AAG was also monitored on single-cell imaging using the LV200 system (Figure 4B and C). Similarly, neither treatment induced activation of the Hsp70 promoter, although 17-AAG induced activation at the single-cell level (Figure 4B and C). These results indicate that treatment with Antp-TPR in the presence of R11-Hsp70 does not affect the transcriptional levels of Hsp70 in cancer cells.

### Effect of treatment with Antp-TPR in the presence of R11-Hsp70 on GSH concentration and ATP dynamics in cancer cells

**Figure 5:**
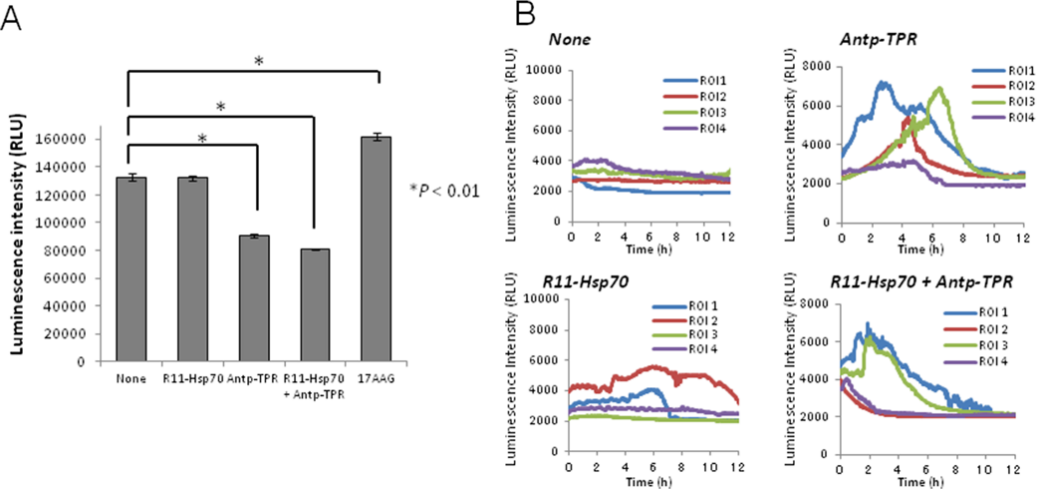
**Treatment with Antp-TPR in the presence of R11-Hsp70 did not increase glutathione (GSH) concentration but affected ATP dynamics in cancer cells. (A)** BT20 cells were treated with or without Antp-TPR (20 μM), R11-Hsp70 (10 μM), or a combination of these peptides for 24 h and subjected to a GSH assay using a luminometer as described in the Materials and Methods section. A total of 0.5 μM 17-AAG was used as a positive control for the increase in GSH concentration in cancer cells. Data are expressed as the means ± SD. **(B)** BT20 cells were transiently transfected with a pGL4.50 vector and subjected to time-course analysis of ATP dynamics on single-cell imaging using a LV200 system after treatment with or without Antp-TPR, R11-Hsp70, or a combination of these peptides.

Treatment with 17-AAG was previously reported to induce an increase in GSH concentration, via up-regulation of Hsp27, and may therefore have the potential to affect the sensitivity of cancer cells to not only this compound but also other anticancer agents [26]. In this study, however, treatment of cancer cells with Antp-TPR in the presence of R11-Hsp70 did not induce up-regulation of Hsp27 protein (Figure 3). These findings prompted us to examine the effect of on GSH concentration in cancer cells of treatment with Antp-TPR in the presence of R11-Hsp70. As shown in Figure 5A, Antp-TPR with or without R11-Hsp70 did not increase the GSH concentration in cancer cells, whereas 17-AAG did. When we examined ATP dynamics in cancer cells after treatment with these peptides, single-cell level imaging with the LV200 system revealed that the treatment with Antp-TPR in the presence of R11-Hsp70 affected the dynamics, which was observed after treatment with Antp-TPR alone (Figure 5B).

## Discussion

Treatment with the Antp-TPR hybrid peptide in the presence of R11-Hsp70, a global subcellular target of the Hsp90 and Hsp70 network, provides effective cytotoxic activity toward breast cancer cells (MDA-MB-231, BT20, BT474, and MDA-MB-361). Although the endogenous expression level of Hsp70 in MDA-MB-231 cells was lower than that of other cancer cell lines used in this study (Figure 1B), the effect of combinational treatment of Antp-TPR with R11-Hsp70 on cytotoxic activity was almost same level with other cell lines (Figure 1A). It is known that Hsp70 binds to a wide variety of hydrophobic polypeptide sequences in newly synthesized protein and partially folded substrates in the cells [11], and has significant roles to cooperate for protein folding of client proteins which are indispensable for cancer cell growth as mentioned above, in Hsp70/Hsp90 multi-chaperon system [2]. Thus, it is suggested that the inhibition of Hsp70 might exert effectively for cytotoxic activity via the inhibition of Hsp90 even in Hsp70-low expressing cancer cells. It has been reported that BT474 and MDA-MB-361 are Her2 high-expressing, and BT20 and MDA-MB-231 are Her2 low-expressing cancer cells, and that BT20, MDA-MB-361, and BT474 have mutations in *PIK3CA*, and only MDA-MB-231 has *KRAS* mutation among these cell lines [27, 28]. It has been also reported that BT474 is sensitive to both trastuzumab and lapatinib, and that MDA-MB-361 is sensitive to trastuzumab but resistant to lapatinib [29]. Thus, it was found that the combination treatment of Antp-TPR with R11-Hsp70 could induce effective cytotoxic activity toward breast cancer cell lines used in this study, which have different characteristic such as genetic alterations or sensitivity for molecular targeted drugs. Although the endogenous expression levels of Hsp90 and Hsp70 in MCF-10A cells was not different remarkably compared with that of cancer cell lines except for MDA-MB-231, the expression levels of p53 and Akt was quite different among these cell lines (Figure 1B). It is suggested that the differences for the expression levels of Hsp90 client proteins and enhancement of cellular uptake of Antp-TPR by R11-Hsp70 into MCF-10A compared with that of Antp-TPR by R11-Hsp70 toward cancer cells as described below might contribute to the less cytotoxic activity of Antp-TPR or combinational treatment of Antp-TPR with R11-Hsp70 toward MCF-10A than that of these peptides against cancer cell lines.

When we examined the effect of R11-Hsp70 peptide on the cellular uptake of Antp-TPR by flow cytometry analysis, the increase of fluorescence intensity for Antp-TPR-TAMRA was observed in both cancer and normal cells, and the enhancement of fluorescence intensity toward cancer cells was higher than that of normal cells (Additional file 4). It is suggested that the cellular uptake of Antp-TPR would be enhanced by R11-Hsp70 since it was reported that arginine-rich peptides could significantly contribute against cellular uptake via macropinosytosis [19], which might lead to the increase for cytotoxic activity of Antp-TPR. It was also observed that the enhancement for cellular uptake of Antp-TPR by R11-Hsp70 into these cells was higher than that of Antp-TPR by R11-Hsp70 scramble peptide (Additional file 4). Thus, it is suggested that the sequence of Hsp70 scramble might affect the enhancement for cellular uptake of Antp-TPR by R11 sequence, and as a result, R11-Hsp70 scramble does not increase effectively the cytotoxic activity compared with R11-Hsp70 peptide. Taken together with the result that the combinational treatment of Antp-TPR with R11-Hsp70 was more effectively increased compared with that in the presence of Antp-Hsp70 toward cancer cells as mentioned above, it is also suggested that the combination of Antp-TPR with R11-Hsp70 would exert to the effective cellular uptake of Antp-TPR. Specifically, Antp-TPR in the presence of R11-Hsp70 synergetically enhanced cytotoxic activity causing effective decrease of Hsp90 client proteins such as Akt, p53, and cRaf in the cytosol, and interestingly, the increase of several K48-linked-polyubiqutin proteins located at approximate 40–48 kDa was also observed (Additional file 3), suggesting that the combinational treatment of Antp-TPR with R11-Hsp70 may cause the change of environment and protein homeostasis in cancer cells, although these increased proteins are still unidentified and further study would be needed to reveal the mechanism caused by these peptides.

Since it is known that breast cancer is a clinically heterogeneous disease with multiple genetic abnormalities, targeting and inhibiting Hsp90 has been considered an effective strategy for breast cancer therapy [5]. Thus, Hsp90 inhibitors have been interesting targets in the quest to improve breast cancer treatment [5]. However, the clinical application of current Hsp90 inhibitors has resulted in only small gains in cancer patients with several problems [30]. In this study, we confirmed the diverse molecular activity of Antp-TPR in the presence of R11-Hsp70, compared with 17-AAG, toward cancer cells and we found that the combination of Antp-TPR with R11-Hsp70 induced neither up-regulation of Hsp90, Hsp70, Hsp27, or Hop, nor an increase in GSH concentration in breast cancer cells (Figure 3, Figure 4, and Figure 5A). Conventional Hsp90 inhibitors such as 17-AAG induce up-regulation of heat shock proteins, including Hsp90, Hsp70, and Hsp27, that likely correlates with decreased anticancer activity [24, 25]. Furthermore, treatment with 17-AAG is reported to increase the concentration of GSH in cancer cells, which can cause resistance to this compound [26]. It was found that Antp-TPR with or without R11-Hsp70 decreased the GSH concentration in cancer cells (Figure 5A). Since Antp-TPR caused the rapid cancer cell-killing compared with 17-AAG, and 17-AAG induced the up-regulation of Hsp proteins quickly as reported previously [14], and neither Antp-TPR alone nor Antp-TPR with R11-Hsp70 induced the up-regulation of Hsp proteins as mentioned above, it is suggested that the disruption of protein homeostasis without the up-regulation of Hsp proteins through inhibition of Hsp90 or Hsp90 and Hsp70 function by Antp-TPR with or without R11-Hsp70 might affect the GSH concentration in cancer cells. Thus, treatment of cancer cells with these peptides, which does not increase the concentration of GSH, might have an additional advantage over current Hsp90-targeted small compounds such as 17-AAG, although the effective increase in cytotoxic activity of paclitaxel toward cancer cells was not observed (data not shown) when we tested the effect of combinational treatment of Antp-TPR with R11-Hsp70 on the cytotoxic activity of this anti-cancer drug, which is one of the often used anti-cancer drugs clinically, suggesting that the decrease effect of GSH by these peptides in cancer cells might not be so significant for the cytotoxic activity of anti-cancer drugs.

Since our previous study showed that bioluminescence imaging at the single-cell level with the LV200 imaging system is a powerful tool for analyzing gene expression (based on a reporter assay specific to cells with low transfection efficiency) [23], we applied this imaging technique to evaluate the effect of each treatment on Hsp70 expression. Although the transfection efficiency of reporter plasmid DNA in BT20 was not so high compared with that of other cancer cells with high transfection efficiency, such as HeLa cells [23], analyzing Hsp70 promoter activation on single-cell imaging proved to be effective for time-course monitoring of this promoter in living cancer cells. Treatment with Antp-TPR with or without R11-Hsp70 did not activate the Hsp70 promoter in breast cancer cells, whereas treatment with 17-AAG did (Figure 4C). Thus, combining bioluminescence imaging at the single-cell level with conventional experiments will be useful for not only promoter analysis but also for evaluating or predicting resistance to anticancer drugs. We also examined the ATP dynamics in cancer cells using the LV200 system after treatment with Antp-TPR in the presence or absence of R11-Hsp70 in this study, since it was reported that elevated cytosolic ATP level can serve as one of indicators of apoptotic cell death [31]. Antp-TPR elevated the ATP levels in cancer cells, which is coincident with the findings of our previous study involving flow cytometry analysis with annexin V staining [13], while treatment with Antp-TPR in the presence of R11-Hsp70 shifted the ATP dynamics compared with treatment with Antp-TPR alone (Figure 5B). However, the increase in Annexin V positive cells was not observed, and PI positive cells were increased in the combinational treatment with these peptides compared with Antp-TPR alone (Additional file 5) when we performed the flow cytometry analysis using Annexin V and PI staining. Thus, it is suggested that the acceleration of ATP increase in cancer cells shown by LV200 might be caused by the more rapid and necrosis-like cell death in the combinational treatment of Antp-TPR with R11-Hsp70 than that of Antp-TPR alone as shown in Figure 2A.

We previously reported that the cytotoxic activity of Antp-TPR toward cancer cells was not affected in the presence of the Hsp70-specific inhibitor 2-phenylethynesulfonamide, although the cytotoxic activity of 17-AAG was increased under this condition [14]. However, when we examined the effect of another Hsp70 inhibitor, VER155008, the cytotoxic activity of Antp-TPR toward cancer cells was increased, whereas VER155008 did not have any synergetic effect on the cytotoxic activity of 17-AAG under the same conditions (Additional file 6). It was recently reported that VER155008 could bind to the nucleotide binding site of Hsp70 protein, and then could act as ATP-competitive inhibitor, which prevents allosteric control between nucleotide binding domain and substrate binding domain, although 2-phenylethynesulfonamide interacted with the substrate binding domain of Hsp70 in an unspecific and detergent-like fashion [32]. In addition, VER155008 more effectively inhibited the refolding and ATPase activities of Hsp70 compared with 2-phenylethynesulfonamide [32]. It was also recently reported that 2-phenylethynesulfonamide caused the environment of lysosome permeabilization and cathepsin D release from lysosomes in lymphoma cells [33]. Taken together with these recent repots, it is suggested that the combination treatment of Antp-TPR with VER155008 might be better for increase of the cytotoxic activity than the combination with 2-phenylethynesulfonamide. On the other hand, the unspecific hydrophobic interaction of 17-AAG with VER155008 or the decrease of solubility in the solution might occur and affect the cytotoxic activity of 17-AAG because of the hydrophobicity of these compounds, although further investigations would be needed for the elucidation of these suggestions. Thus, further study about the combination of Antp-TPR with other peptides or small compounds targeting Hsp70 may lead to significant improvements in the cytotoxic activity of Antp-TPR toward cancer cells.

## Conclusion

The combination of Antp-TPR and an Hsp70-targeted peptide has sufficient molecular features for targeting both Hsp90 and Hsp70. Such activity can induce synergetic cytotoxic activity toward cancer cells by simultaneously decreasing multiple Hsp90 client proteins, providing a potent advantage over conventional Hsp90 inhibitors such as 17-AAG. Taken together, targeting Hsp90 with the Antp-TPR hybrid peptide might lead to a new therapeutic approach for managing malignant human tumors, including breast cancer. Thus, the present findings will assist in further development of cancer treatments targeting Hsp90 and Hsp70.

## Electronic supplementary material

Additional file 1: **Effect of R11-heat shock protein (Hsp) 70 peptide on the cytotoxic activity of Antp-TPR or 17-allylamino-demethoxygeldanamycin (17-AAG) in cancer and normal cells.** **(A)** Viability of MDA-MB-231 and BT20 cells treated with Antp-TPR, R11-Hsp70, or a combination of these peptides. Cells were incubated with Antp-TPR, R11-Hsp70, or Antp-TPR in the presence of R11-Hsp70 (10 μM) at the indicated concentrations for 24 h and analyzed for cell viability as described in the Materials and Methods section. **(B)** Viability of MDA-MB-231 and BT20 cells treated with Antp-TPR in the presence or absence of R11-Hsp70scramble. Cells were incubated with Antp-TPR or Antp-TPR in the presence of R11-Hsp70scramble (10 μM) at the indicated concentrations for 24 h and analyzed for cell viability. **(C)** Normal mammary epithelial cells, MCF-10A (left) or BT20 (right) cells were incubated with Antp-TPR in the presence or absence of R11-Hsp70 (10 μM), or 17-AAG in the presence or absence of R11-Hsp70 (10 μM), respectively, at the indicated concentrations for 24 h and analyzed for cell viability. Data are expressed as the means ± SD. (TIFF 112 KB)

Additional file 2: **Effect of high concentration of R11-Hsp70 on the expression levels of Hsp90, Hsp70, Akt, and p53 proteins.** BT20 and MDA-MB-231 cells were treated with or without R11-Hsp70 (40 μM) for 18 h and examined by western blotting for the expression of Hsp90, Hsp70, Akt, p53, and β-actin using corresponding antibodies. β-Actin was used as the loading control. Bands were visualized by chemiluminescence as described in the Materials and Methods section. (TIFF 101 KB)

Additional file 3: **Detection of polyubiquitinylated proteins after treatment with Antp-TPR in the presence of R11-Hsp70 peptide.** BT20 cells were treated with or without Antp-TPR, R11-Hsp70, or a combination of these peptides for the indicated times and examined by western blotting for polyubiquitinylated proteins using anti-polyubiquitinylated protein and anti-K48-linkage-specific polyubiquitin antibodies. β-Actin was used as the loading control. All bands were visualized by chemiluminescence. Asterisk (*) indicates the location of increased K48-linkaged-polyubiquitin proteins by the combinational treatment of Antp-TPR with R11-Hsp70. (TIFF 547 KB)

Additional file 4: **Effect of R11-Hsp70 on the cellular uptake of Antp-TPR peptide for cancer or normal cells.** (A) BT20, MDA-MB-231, or MCF-10A cells were incubated with or without Antp-TPR-TAMRA (10 μM) in the presence or absence of R11-Hsp70 (10 μM) or R11-Hsp70 scramble peptide (10 μM) for 30 min, and then flow cytometry assay was performed as described in the Materials and Methods section. Upper graphs indicate the fold of fluorescence intensity obtained from the results of histograms (lower panels). (B) Internalization of R11-Hsp70 peptide toward BT20, MDA-MB-231, or MCF-10A cells was also confirmed after the treatment of these cells with R11-Hsp70-FITC (10 μM) for 30 min by flow cytometry assay. (TIFF 242 KB)

Additional file 5: **Flow cytometry analysis by Annexin V and PI staining.** BT20 cells were treated with or without Antp-TPR (20 μM) in the presence or absence of R11-Hsp70 (10 μM) for 2 hr, and then flow cytometry analysis by either Annexin V (A) or PI (B) staining alone, or Annexin V and PI (C) staining was performed as described in the Materials and Methods section. The numbers in graphs indicate the percentage of cells in each quadrant. (TIFF 324 KB)

Additional file 6: **Effect of Hsp70 inhibitor on the cytotoxic activity of Antp-TPR or 17-AAG toward BT20 cells.** BT20 cells were treated with Antp-TPR or 17-AAG at the indicated concentrations in the presence or absence of 5 μM VER155008 and subjected to the WST-8 assay for the assessment of cell viability. Data are expressed as the means ± SD. (TIFF 67 KB)

## Abbreviations

Hsp90: Heat shock protein 90
Hop: p60/Hsp-organizing protein
TPR: Tetratricopeptide repeat
Antp: Antennapedia homeodomain sequence
IC50: The peptide concentration inducing 50% inhibition of control cell growth
TAMRA: Carboxytetramethylrhodamine
FITC: Fluorescein isothiocyanate
GSH: Glutathione.
