# Supplementary figures and images for: Synergetic cytotoxic activity toward breast cancer cells enhanced by the combination of Antp-TPR hybrid peptide targeting Hsp90 and Hsp70-targeted peptide
Source: BMC Cancer. 2014 Aug 26;14:615. doi: 10.1186/1471-2407-14-615 (PMC4153893; doi:10.1186/1471-2407-14-615)

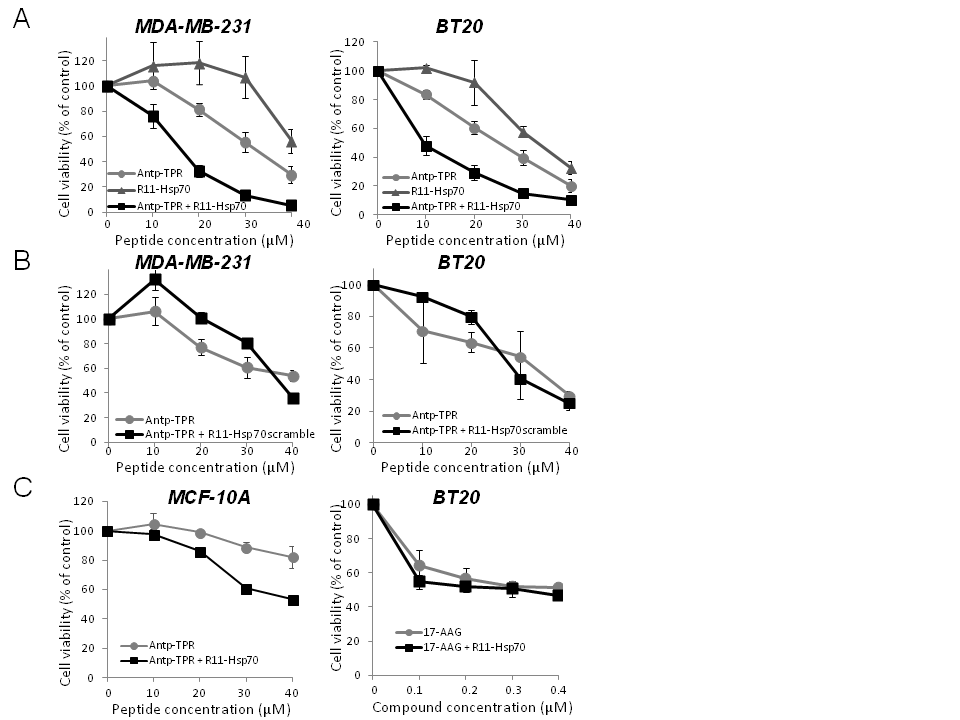

Supplement: Supplementary file 1 — Additional file 1: Effect of R11-heat shock protein (Hsp) 70 peptide on the cytotoxic activity of Antp-TPR or 17-allylamino-demethoxygeldanamycin (17-AAG) in cancer and normal cells. (A) Viability of MDA-MB-231 and BT20 cells treated with Antp-TPR, R11-Hsp70, or a combination of these peptides. Cells were incubated with Antp-TPR, R11-Hsp70, or Antp-TPR in the presence of R11-Hsp70 (10 μM) at the indicated concentrations for 24 h and analyzed for cell viability as described in the Materials and Methods section. (B) Viability of MDA-MB-231 and BT20 cells treated with Antp-TPR in the presence or absence of R11-Hsp70scramble. Cells were incubated with Antp-TPR or Antp-TPR in the presence of R11-Hsp70scramble (10 μM) at the indicated concentrations for 24 h and analyzed for cell viability. (C) Normal mammary epithelial cells, MCF-10A (left) or BT20 (right) cells were incubated with Antp-TPR in the presence or absence of R11-Hsp70 (10 μM), or 17-AAG in the presence or absence of R11-Hsp70 (10 μM), respectively, at the indicated concentrations for 24 h and analyzed for cell viability. Data are expressed as the means ± SD. (TIFF 112 KB) [file 12885_2013_4800_MOESM1_ESM.tiff]

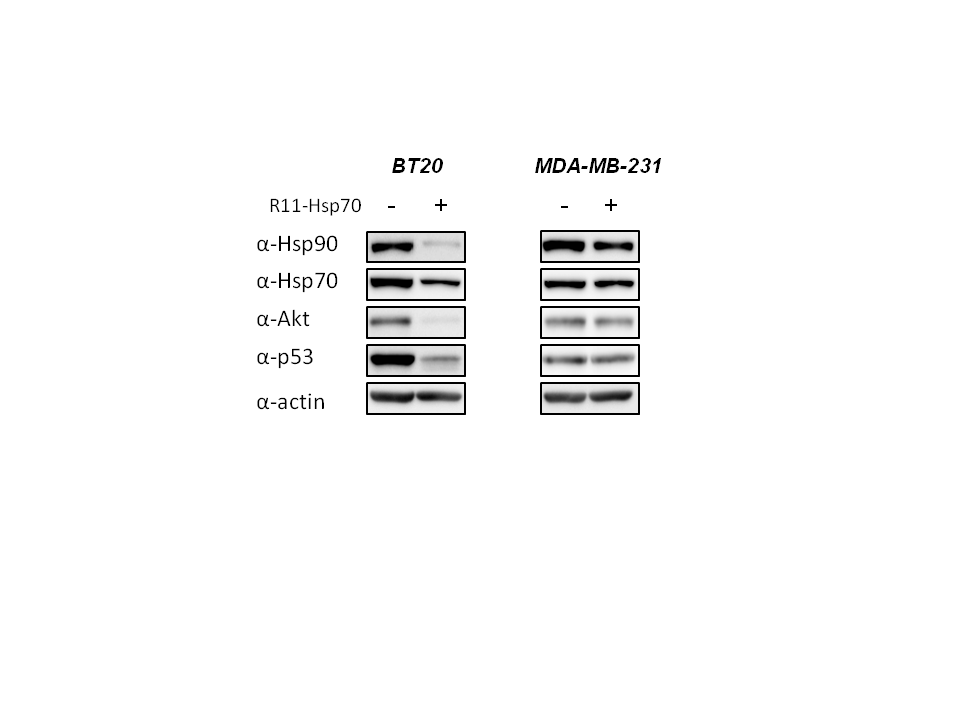

Supplement: Supplementary file 2 — Additional file 2: Effect of high concentration of R11-Hsp70 on the expression levels of Hsp90, Hsp70, Akt, and p53 proteins. BT20 and MDA-MB-231 cells were treated with or without R11-Hsp70 (40 μM) for 18 h and examined by western blotting for the expression of Hsp90, Hsp70, Akt, p53, and β-actin using corresponding antibodies. β-Actin was used as the loading control. Bands were visualized by chemiluminescence as described in the Materials and Methods section. (TIFF 101 KB) [file 12885_2013_4800_MOESM2_ESM.tiff]

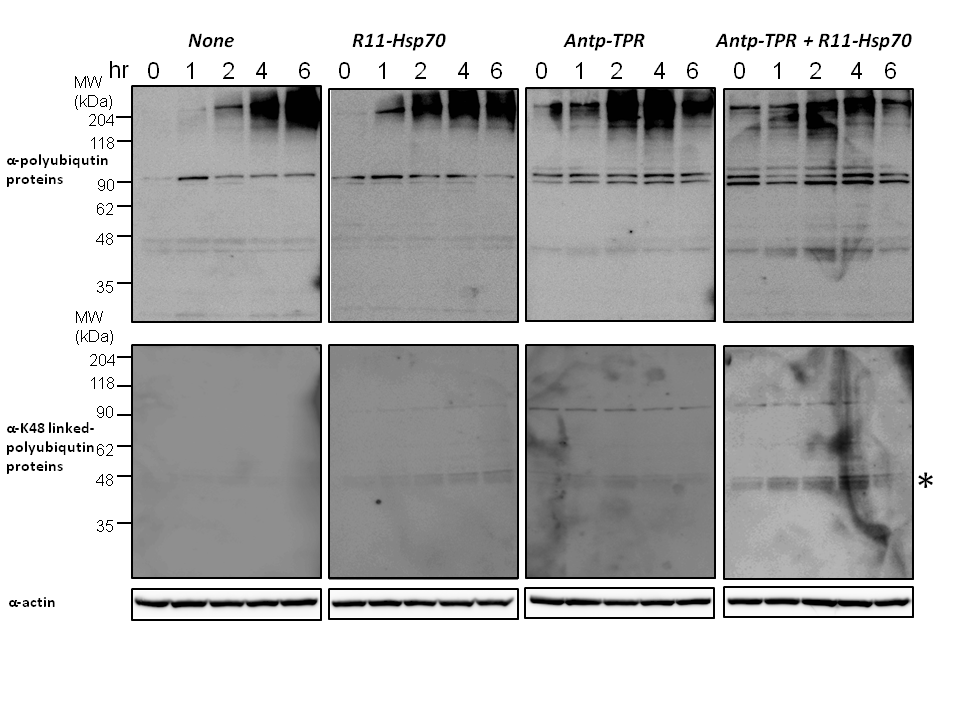

Supplement: Supplementary file 3 — Additional file 3: Detection of polyubiquitinylated proteins after treatment with Antp-TPR in the presence of R11-Hsp70 peptide. BT20 cells were treated with or without Antp-TPR, R11-Hsp70, or a combination of these peptides for the indicated times and examined by western blotting for polyubiquitinylated proteins using anti-polyubiquitinylated protein and anti-K48-linkage-specific polyubiquitin antibodies. β-Actin was used as the loading control. All bands were visualized by chemiluminescence. Asterisk (*) indicates the location of increased K48-linkaged-polyubiquitin proteins by the combinational treatment of Antp-TPR with R11-Hsp70. (TIFF 547 KB) [file 12885_2013_4800_MOESM3_ESM.tiff]

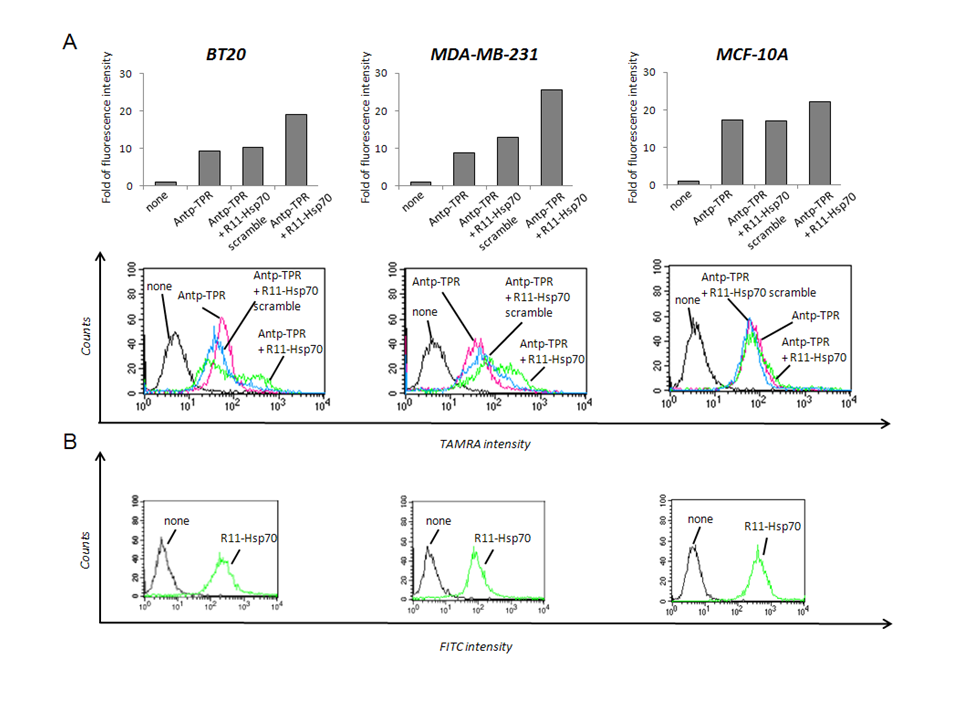

Supplement: Supplementary file 4 — Additional file 4: Effect of R11-Hsp70 on the cellular uptake of Antp-TPR peptide for cancer or normal cells. (A) BT20, MDA-MB-231, or MCF-10A cells were incubated with or without Antp-TPR-TAMRA (10 μM) in the presence or absence of R11-Hsp70 (10 μM) or R11-Hsp70 scramble peptide (10 μM) for 30 min, and then flow cytometry assay was performed as described in the Materials and Methods section. Upper graphs indicate the fold of fluorescence intensity obtained from the results of histograms (lower panels). (B) Internalization of R11-Hsp70 peptide toward BT20, MDA-MB-231, or MCF-10A cells was also confirmed after the treatment of these cells with R11-Hsp70-FITC (10 μM) for 30 min by flow cytometry assay. (TIFF 242 KB) [file 12885_2013_4800_MOESM4_ESM.tiff]

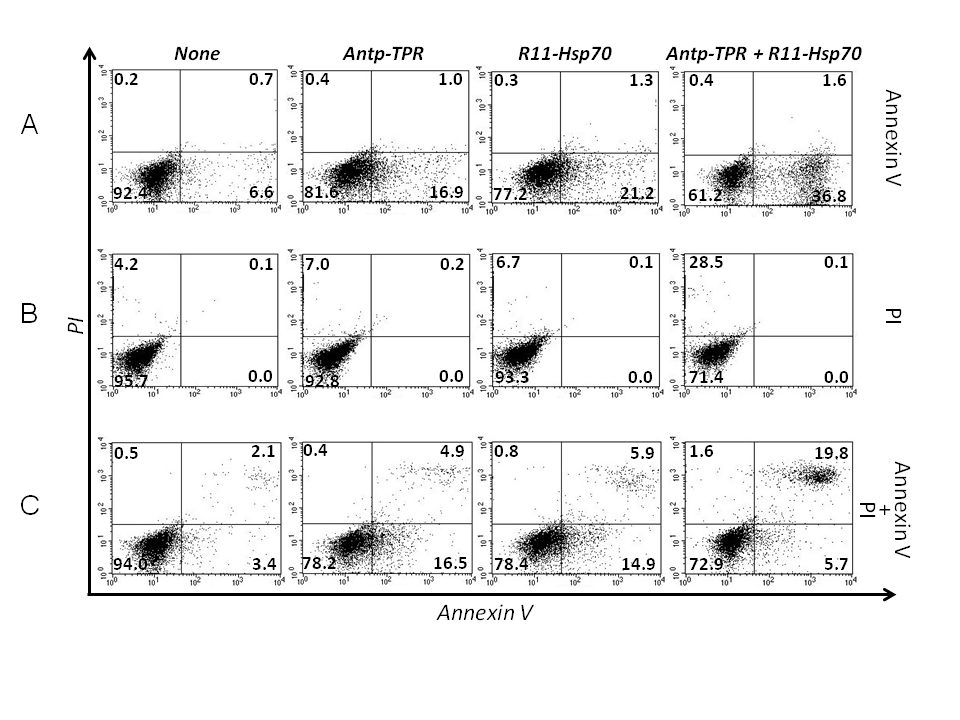

Supplement: Supplementary file 5 — Additional file 5: Flow cytometry analysis by Annexin V and PI staining. BT20 cells were treated with or without Antp-TPR (20 μM) in the presence or absence of R11-Hsp70 (10 μM) for 2 hr, and then flow cytometry analysis by either Annexin V (A) or PI (B) staining alone, or Annexin V and PI (C) staining was performed as described in the Materials and Methods section. The numbers in graphs indicate the percentage of cells in each quadrant. (TIFF 324 KB) [file 12885_2013_4800_MOESM5_ESM.tiff]

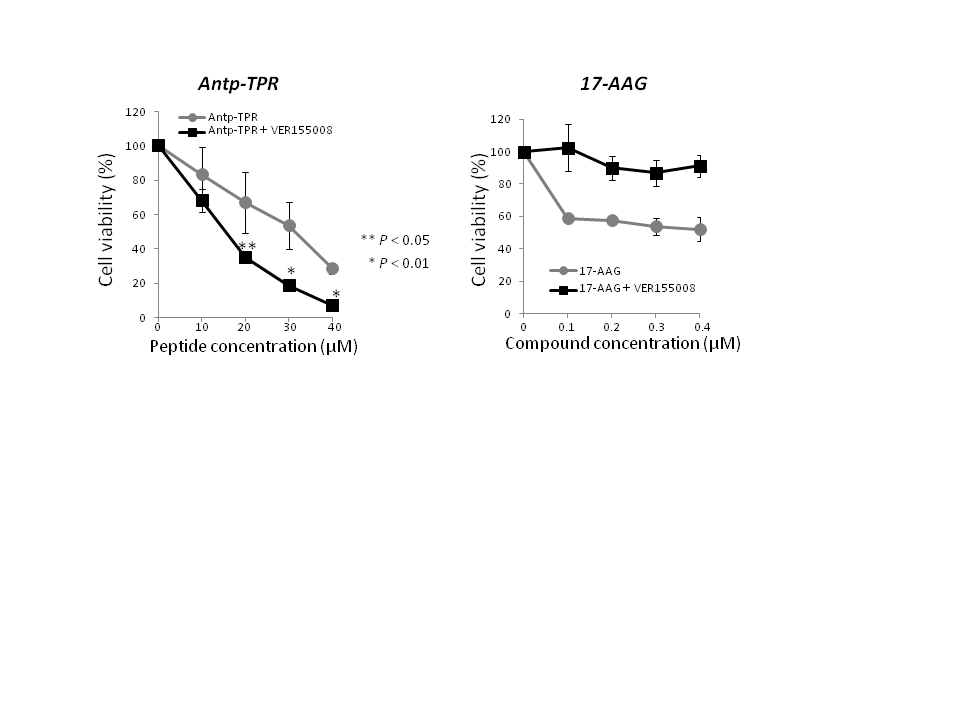

Supplement: Supplementary file 6 — Additional file 6: Effect of Hsp70 inhibitor on the cytotoxic activity of Antp-TPR or 17-AAG toward BT20 cells. BT20 cells were treated with Antp-TPR or 17-AAG at the indicated concentrations in the presence or absence of 5 μM VER155008 and subjected to the WST-8 assay for the assessment of cell viability. Data are expressed as the means ± SD. (TIFF 67 KB) [file 12885_2013_4800_MOESM6_ESM.tiff]
